# Antimetastatic and Antitumor Activities of Orally Administered NAX014 Compound in a Murine Model of HER2-Positive Breast Cancer

**DOI:** 10.3390/ijms22052653

**Published:** 2021-03-06

**Authors:** Elisa Pierpaoli, Francesco Piacenza, Gaetano Fiorillo, Paolo Lombardi, Fiorenza Orlando, Carmela Salvatore, Cristina Geroni, Mauro Provinciali

**Affiliations:** 1 Advanced Technology Center for Aging Research, Scientific Technological Area, IRCCS INRCA, 60121 Ancona, Italy; e.pierpaoli@inrca.it (E.P.); m.provinciali@inrca.it (M.P.); 2 Naxospharma Srl, 20026 Novate Milanese, Italy; gtnfiorillo@gmail.com (G.F.); p.lombardi@naxospharma.eu (P.L.); 3 Experimental Animal Models for Aging Unit, Scientific Technological Area, IRCCS INRCA, 60121 Ancona, Italy; f.orlando@inrca.it; 4 Aesis Therapeutics Srl, Incubatore JCube, 60035 Jesi, Italy; salvatore.carmela@libero.it (C.S.); cristina.geroni@hotmail.it (C.G.)

**Keywords:** berberine, NAX014, breast cancer, HER-2, lung metastases, senescence, *p16*, TNF-α, VEGF, migration

## Abstract

The natural isoquinoline alkaloid Berberine (BBR) has been shown to possess several therapeutic effects, including anticancer activity. Different BBR derivatives have been designed and synthesized in order to obtain new compounds with enhanced anticancer efficacy. We previously showed that intraperitoneal (IP) administration of the BBR-derived NAX014 compound was able to counteract HER-2 overexpressing mammary tumors onset and progression in transgenic mice. However, the IP administration was found to induce organ toxicity at doses higher than 2.5 mg/Kg. In this study, we evaluated the effect of intragastric (IG) administration of 20 mg/kg of NAX014 on both safety and anticancer efficacy in HER-2/neu transgenic mice. Furthermore, cancer cell dissemination and migration, tumor cell senescence and immunological changes were examined. Our results demonstrated that IG NAX014 administration delayed the onset of mammary tumors with no negative effects on health and survival. NAX014 reduced HER-2 overexpressing BC cells migration in vitro and the frequency of lung metastasis in HER-2/neu transgenic mice. A statistically significant increase of senescence-associated *p16* expression was observed in tumors from NAX014-treated mice, and the induction of cell senescence was observed in HER-2 overexpressing BC cells after in vitro treatment with NAX014. Although NAX014 did not modulate the presence of tumor-infiltrating lymphocytes, the level of circulating TNF-α and VEGF was found to be reduced in NAX014-treated mice. The overall results address the NAX014 compound as potential tool for therapeutic strategies against HER-2 overexpressing breast cancer.

## 1. Introduction

Breast cancer (BC) is the most frequent cancer in women worldwide and the second leading cause of cancer-related deaths in women. The human epidermal growth factor receptor 2 (HER-2) is over-expressed in 15–20% of invasive BCs and is associated with aggressive phenotype, lower survival rate and higher risk for recurrent disease after primary therapy. The advent of HER-2 targeted therapies (i.e., trastuzumab, pertuzumab) has been shown considerable improvements in the outcomes of patients with HER-2 overexpressing BC [1]. However, issues related to drug resistance and toxicity significantly limits their safe usage in cancer treatment [2,3]. In this context, emerging therapies and promising alternative approach of BC management, are under investigation [4,5].

Several plant-derived compounds have gained widespread attention for their role in inhibiting cancer progression and overcoming drug resistance. Berberine (BBR) is an isoquinoline alkaloid isolated from the roots and bark of several herbs. The earliest evidence of pharmaceutical properties of BBR dates back to 1930 and reports an efficacy protocol of oriental sore treatment with local injection of BBR acid sulphate [6]. Subsequently, a long series of studies highlighted the wide spectrum of BBR effects covering anti-cancer, anti-infective and immune-modulating activities [7]. Moreover, in the last decades, in order to improve the BBR activity, bioavailability and efficacy, several BBR-derived compounds have been also successfully developed [8,9].

It has been previously shown that introduction of the specific chemical modification in BBR, represented by an aromatic moiety bonded to the 13-position of parent alkaloid through a linker of variable length, considerably improves its anticancer effect on, inter alia, breast, colon, and pancreatic cancer cells [10,11,12]. We found in an in vitro model that the presence of the electron-withdrawing group Cl on the aromatic moiety linked to NAX014 was able to exert a higher anti-proliferative effect on HER-2 overexpressing Breast Cancer (BC) cells with respect to that one brought by BBR [8]. NAX014 showed also a higher anticancer activity with respect to the unsubstituted analogs NAX012, NAX035, and NAX013 (which conversely bears an electron-donating group), by inducing apoptosis, cellular senescence, and reducing both HER-2 expression and phosphorylation [8].

In in vivo model, intraperitoneal (IP) administration of NAX014 was able (a) to delay the development of mammary tumors, (b) to reduce the tumor volume and tumor vessel density and (c) to increase tumor cell senescence in HER-2/neu transgenic mice [11]. In this experimental model, the dosage of NAX014, and BBR in a higher extent, was limited because of increasing organ injury and mortality rate with doses higher than 2.5 mg/Kg, as observed during the toxicity study [11]. In particular, the IP administration of 10 mg/Kg BBR and 20 mg/Kg NAX014 caused 75% and 25% of animal deaths, respectively, within four weeks from the first administration.

It has been previously shown that BBR toxicity and bioavailability change according to the route of administration [13]. Although the bioavailability of BBR by oral administration was lower than that by IP, no signs of toxicity were found at oral dosage 360 times higher than that of IP [13]. These findings suggested further experiments with NAX014 oral administration, considering also that the IP administration route is a common technique in laboratory rodents, but rarely is used in humans.

Therefore, in this study we attempted to evaluate the effect of intragastric (IG) administration of 20 mg/kg of NAX014 compound on safety, antitumor and antimetastatic activities as evaluated by the kinetic of development of spontaneous mammary tumors and lung metastases in HER-2/neu transgenic mice. HER-2 expression was determined to control the tumorigenesis on the mammary gland whereas TNF-α and VEGF plasma levels were determined to evaluate both inflammation and angiogenesis. The effect of NAX014 on cell migration/invasion and induction of cell senescence was evaluated both in vitro and in vivo confirming their role in the inhibition of tumorigenesis.

## 2. Results

**Figure 1:**
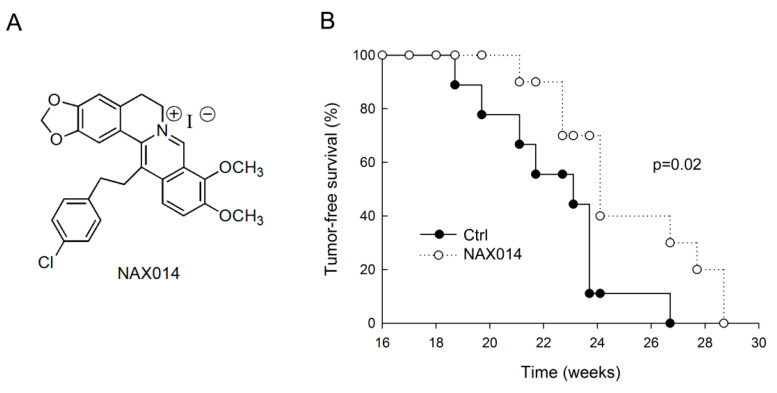
Effect of IG NAX014 administration on the kinetics of tumor growth in HER-2/neu transgenic mice. (**A**) Chemical structure of NAX014. (**B**) FVB/N HER-2/neu transgenic mice (*n* = 10) were intragastrically administered two times a week with 20 mg/kg b.w. of NAX014 or vehicle alone (Ctrl) and analyzed for tumor incidence. The percentage of tumor-free mice was calculated as the cumulative number of mice with tumors and mice that were tumor-free. Kaplan–Meier analysis was performed to generate the tumor-free curves. Statistical significance (*p*) is reported in the text.

We evaluated the effect of IG administration of the BBR-derived NAX014 compound (Figure 1A) on spontaneous mammary carcinogenesis in HER-2/neu transgenic mice. Starting from the 16th week of age, mice were intragastrically administered twice a week with 20 mg/Kg b.w. of NAX014. The administration was well tolerated, with no effects on mice survival, no evident signs of toxicity and no significant effect on body weight in any group. As shown in Figure 1B, the first mammary tumor appeared in control mice at 18.7 weeks of age followed by NAX014-treated mice at 21.1 weeks of age. In NAX014 group the kinetics of appearance of mammary tumors (tumor-free) was significantly different when compared with control group (*p* = 0.02). Differences in mean tumor number and volume in NAX014-treated mice compared with control group were not found (Figure S1).

**Figure 2:**
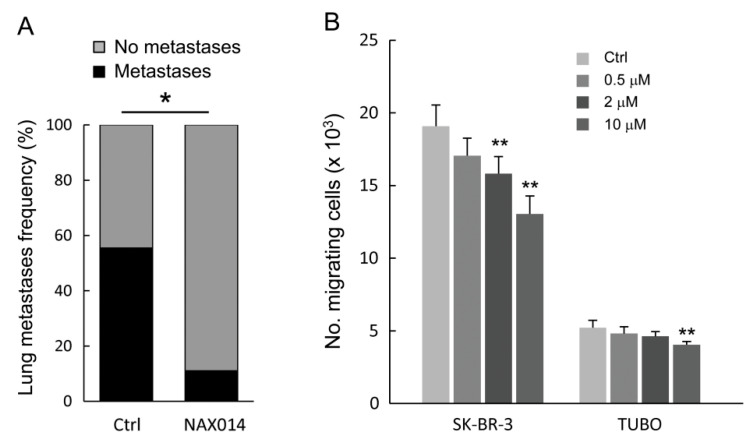
Effect of NAX014 on in vivo lung metastasis and in vitro cell migration. (**A**) Lungs from euthanized FVB/N HER-2/neu transgenic mice were excised and the presence of metastases was evaluated after staining by endotracheal infiltration with 15% china ink solution. Data are reported as percentage of mice with lung metastases for each group. * *p* ≤ 0.05 versus control group. (**B**) Human SK-BR-3 and murine TUBO cell lines were pre-treated with NAX014 (0.5, 2 and 10 μM) for 48 h. The number of migrated cells was counted. Results of quantitative assessment are shown as the mean ± SD from four independent wells. Graph is representative of two independent experiments. ** *p* ≤ 0.05 vs. control cells treated with vehicle.

As shown in Figure 2A, NAX014 administration greatly and significantly reduced the frequency of lung metastasis compared to control mice. Mice bearing lung metastases were 11.1% and 55.5% in NAX014 and control groups, respectively (*p* = 0.033). No differences in mean values of both size and number of lung metastases per mouse have been found. To test whether NAX014 can reduce breast cancer cell migration and invasion, the HER-2 overexpressing human SK-BR-3 and murine TUBO cell lines were treated with low doses of NAX014 (0.5, 2, or 10 μM) for 48 h, and subjected to trans-well migration assay. NAX014 dose-dependently inhibited trans-migration of both SK-BR-3 and TUBO cells (Figure 2B). This effect on SK-BR-3 was statistically significant already at a concentration of 2 μM, while TUBO cells showed the inhibitory effect at 10 μM NAX014 when compared to vehicle-treated cells (*p* ≤ 0.05) (Figure 2B).

Ex vivo analyses on tumor masses with comparable volumes were performed to evaluate the expression levels of *HER-2*, *perforins*/*granzyme B* and cell-cycle checkpoint regulators. To evaluate whether the inhibition of mammary tumorigenesis observed in mice treated with NAX014 was due to its effect on mammary gland, we performed real-time PCR analysis for *HER-2* gene expression in mammary tumors. No significant modulation in *HER-2* mRNA expression was observed in tumor tissues from NAX014-treated mice with respect to control.

**Figure 3:**
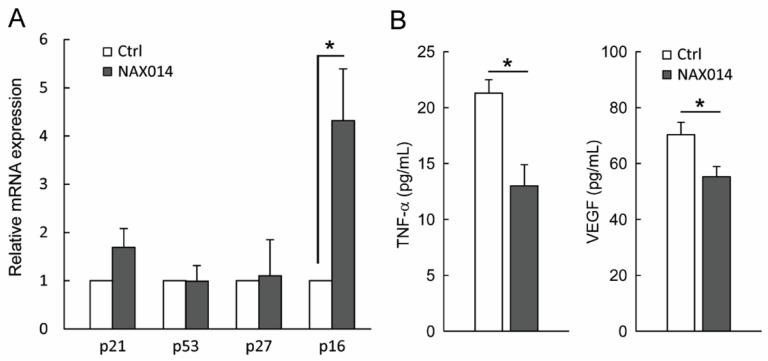
Effect of NAX014 on *p21*, *p53*, *p27* and *p16* expression in mammary tumor samples and on plasma levels of TNF-α and VEGF. (**A**) Relative *p21*, *p53*, *p27* and *p16* mRNAs expression has been analyzed by real time-PCR. mRNA levels are expressed as fold change (ratio of target:reference gene) * *p* ≤ 0.05 versus control values. (**B**) TNF-α and VEGF plasma levels were quantified using ELISA kit. Data are reported as means ± SD. * *p* ≤ 0.05 versus control values.

We thus evaluated the presence of cytotoxic lymphocytes in mammary tumors. To this aim, tumor masses from control and NAX014-treated mice were analyzed for the content of mRNA encoding the cytolytic molecules *perforins* and *granzyme B*. No statistically significant differences between groups have been found (Figure S2). In order to establish whether the effect of NAX014 supplementation on senescent-like growth arrest observed on tumor masses of treated mice could be related to modulation of cell-cycle checkpoint molecules, we measured the mRNA levels of *p21*, *p53*, *p27*, and *p16* in mammary tumor samples. Among the analysed genes, only *p16* showed an increased fold-change >2 in the experimental group with respect to control group (Figure 3A). In this context, a remarkable effect size of the *p16* in the experimental group was found (Cohen’s d = 4.04) (Figure 3A).

Finally, plasma levels of both soluble TNF-α and VEGF in NAX014-treated mice were found to be lower than in control mice (13.0 ± 1.8 pg/mL versus 21.3 ± 1.1 pg/mL and 55.2 ± 3.6 pg/mL versus 70.2 ± 4.4 pg/mL for TNF-α and VEGF respectively; *p* ≤ 0.05) (Figure 3B).

**Figure 4:**
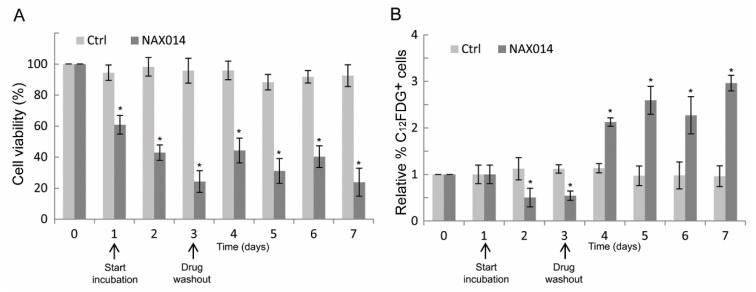
Effect of transient exposure to NAX014 on SK-BR-3 cell viability and cell senescence. SK-BR-3 cells were seeded (day 0) and exposed to 16.9 μM NAX014 (start incubation), or control vehicle (DMSO), for 3 days, after which viable cells were recultured in drug-free medium (drug washout) for additional 4 days, and then analyzed at each time-point for cell viability and cell senescence. (**A**) Cells were counted after trypan blue staining and cell viability was expressed as percentage (%) = (total viable cells (unstained)/total cells (stained and unstained)) × 100. * *p* ≤ 0.05 versus control cells treated with DMSO (**B**). At each time-point, the quantification of cell senescence was assessed by C_12_FDG staining and FACS analysis. Data are presented as the means ± SD of the relative percentage of C_12_FDG-positive cells. * *p* ≤ 0.05 versus control cells treated with DMSO.

To establish whether the effect of NAX014 on the increased *p16* mRNA level observed on tumor masses of treated mice could be related to a senescent-like growth arrest, human SK-BR-3 cells were treated at their corresponding IC_85_ dose of NAX014 (16.9 μM) for three days after which viable cells were cultured in drug-free medium (washout) for additional four days, and then analyzed for cell viability and for the percentage of C_12_FDG-positive senescent cells. As shown in Figure 4A, the viability of NAX014-treated SK-BR-3 cells was reduced, with a rapid decrease after one day (60.8% of viable cells) until reaching a 42.9 and 24.2% of viable cells after two and three days of treatment (*p* ≤ 0.05). Return to NAX014-free medium did not fully recover the cell viability, with 44.2, 31.0, 40.3, and 23.8% of viable cells at four, five, six, and seven days after washout, respectively (Figure 4A). The percentage of C_12_FDG-positive cells decreased after two and three days of NAX014 treatment (0.50 and 0.54% of C_12_FDG+ cells, respectively) (Figure 4B). A statistically significant persistent increase of senescent C_12_FDG+ cells was observed after NAX014 washout, with 2.12, 2.59, 2.27, and 2.96% of C_12_FDG+ cells, at four, five, six, and seven days after washout, respectively (*p* ≤ 0.05) (Figure 4B).

## 3. Discussion

Currently, a considerable number of natural products or direct derivatives of natural products are used in cancer therapy [14]. In vitro anticancer activity of BBR has been extensively demonstrated [7]. However, issues related to BBR poor bioavailability and toxicity may limit its translation to clinical trials. New compounds obtained by chemical manipulation of the parental molecule provide a source of promising chemotherapeutic or chemo-preventive agents [15,16,17]. Nevertheless, preclinical evidences of antitumor efficacy of BBR-derivatives are scarce or absent for most of them. In order to improve the relevance of the BBR-derived NAX014 compound as anticancer drug in preclinical models, we evaluated both the safety and the therapeutic efficacy of NAX014 through oral (intragastric, IG) administration in HER-2/neu transgenic mice.

The present study showed that IG NAX014 administration (i) delays the development of spontaneous mammary tumors in HER-2/neu transgenic mice without negative effects on health and survival; (ii) inhibits breast cancer cell migration and metastasis in vitro and in vivo; (iii) induces in vitro and in vivo tumor cells senescence; and (iv) decreases TNF-α and VEGF plasma levels.

We previously showed that NAX014 is more effective than parental compound (BBR) in exerting anticancer activity in a murine model of spontaneous HER-2/neu breast cancer when administered via IP injection [11]. In that model the IP administration was found to induce organ toxicity at doses higher than 2.5 mg/Kg with 25% of mortality observed after subchronic IP administration of 20 mg/Kg NAX014. In the present study, no deaths have been recorded during IG administration of the same dose, confirming 20 mg/Kg a safe dose. Indeed, mice were IG-administered twice a week for approximately 2–3 months before euthanasia and no signs of toxicity and no changes in body weight were observed during this period, confirming the dosage was safe for mice and potentially translational to humans. Our results agree with data previously reported on the safety of BBR per os administration [13].

The route of administration is often related to physicochemical properties of the drug. The development of cancer drugs that can be administered efficaciously through an oral method is becoming increasingly common because of several advantages, including convenience in synthesis and administration, lower risk of severe toxicity and better patient compliance [18]. The possibility to administer the NAX014 agent through oral method, with an extended activity over the primary tumor to the lung metastases in comparison with the intraperitoneal method, considerably improves its relevance as new potential therapeutic tool in the treatment of HER-2 positive breast cancer.

One of the most relevant results of this study is related to the capability of NAX014 compound to inhibit in vivo distant metastases formation. In this study, IG NAX014 treatment strongly reduced the incidence of lung metastases when compared to control mice. Trans-well migration assay confirmed the effect of NAX014 on suppressing the cancer cell migration of HER-2 overexpressing BC cell lines. In particular, results from the TUBO cell line, established from transgenic BALB/c mice carrying the rat HER-2/neu proto-oncogene under MMTV promoter, are noteworthy, because strictly reflecting the experimental animal model we used. Lung metastasis is one of the most common distant metastases of breast cancer and is associated with poor prognosis [19]. Previous studies have reported that the recurrence probability of distant metastases in patients with HER-2 positive was higher than HER-2 negative tumors [20]. Moreover, older adult patients with HER-2 positive tumors were more likely to develop lung metastases [21]. In this context, this study suggests the implementation of NAX014 in other cancer models characterized by the development of metastases with advancing age.

The antitumor and antimetastatic effect of NAX014 was associated with decreased plasma level of both vascular endothelial growth factor (VEGF) and tumor necrosis factor-alpha (TNF-α). Inflammation and angiogenesis are dependent processes. VEGF is well known for its role in vasculogenesis and it is thought to contribute to tumor metastasis by promoting both extravasation and tumor angiogenesis [22]. TNF-α is a multifunctional pro-inflammatory cytokine also involved in regulation of normal and pathologic angiogenic processes [23]. Elevation of VEGF and TNF-α from normal levels have been observed in various types of cancer, including BC and have been shown to contribute to tumor angiogenesis and metastasization [24,25]. Therefore, compounds that are able to downregulate these proteins could be considered powerful antiangiogenic and antimetastatic agents. Our findings led to the hypothesis that NAX014 is involved in the regulation of BC metastasization possibly by directly affecting BC cells migration/invasion and by indirectly affecting the secretion of VEGF and TNF-α. In addition, the antimetastatic phenomenon we observed could also be explained through a different degree of tumor vascularization. This hypothesis is in agreement with previous results revealing a significant reduced vessel density in mammary tumors from mice treated with IP 2.5 mg/Kg NAX014 in comparison with control group [11].

Berberine is also known to possess immunosuppressive activity [26]. However, data regarding the immunomodulating effect of NAX014 is still lacking. Here we observed a decreased plasma level of TNF-α in NAX014-treated mice. This result is in line with those found in BBR, which treatment in vitro and in vivo was able to counteract hyper-inflammatory conditions by suppressing the expression of inflammatory mediators such as TNF-α, IL-6, and IL-1β [9,27]. In this context, it is important to remark how the inflammatory component contributes to tumor proliferation, angiogenesis, metastases, and resistance to hormonal and chemotherapy. On the other hand, IG NAX014 administration was not able to produce any modulation on tumor-infiltrating lymphocytes as revealed by mRNA expression analysis of *granzyme B* and *perforin* in tumor masses from control and treated mice. We previously showed a reduction of tumor-infiltrating lymphocytes in IP 2.5 mg/kg NAX014-treated mice which was attributed to the reduced tumor vascularization [11]. The result of the present study suggests a comparable degree of vascularization between IG NAX014-treated and control mice. Further studies need to be performed in order to clarify the relationship between vascularization and tumor progression.

As previously observed in IP NAX014-treated mice [11], IG NAX014 administration was able to induce cell senescence in tumor masses, as revealed by increased in situ expression of tumor suppressor *p16*. The in vitro β-galactosidase assay on HER-2 overexpressing SK-BR-3 cells demonstrated the involvement of cellular senescent-like pathways in the NAX014-mediated antitumor effects. In our experiments, the NAX014 treatment induced severe cytotoxicity followed by a weak recovery of cell viability, and a massive increase of the number of senescent cells during NAX014-free recovery. These results agree with previous findings showing the induction of apoptotic cell death and senescence-like growth arrest of HER-2 overexpressing BC cells under treatment with different BBR-derivatives, including NAX014 [8,11,16]. It has been suggested that senescent-like growth arrest may be a significant determinant of tumor response to anticancer agents in conditions in which the induction of cell death does not explain by itself the antiproliferative effect. Although the benefits of cancer cell senescence, in terms of cancer recurrence, are still a topic of debate, a growing number of pro-senescence therapeutic approaches are currently under investigation [28]. At the same time, efforts to develop senolytic compounds that can induce cell death in senescent cells are increasing [29]. Therefore, therapeutic approaches aimed at selectively inducing senescence combined with a second-line senolytic-based adjuvant therapy could represent a promising strategy for cancer treatment. Further studies are needed to explore the efficacy of NAX014 as pro-senescence drug in combination with senolytic-based adjuvant therapy.

In conclusion, this study demonstrated for the first time, the efficacy of the BBR-derivative NAX014 as oral antitumor agent, being effective in counteracting HER-2 positive breast cancer development and spreading in preclinical models. The oral administration offers various advantages in comparison with IP administration, being easier to do, more safe and more effective in its antitumor effect, acting on primary cancer cells as well as on tumor metastases. Further studies on the efficacy and the tolerability are needed to evaluate the possible implementation of NAX014 in clinical trials.

## 4. Materials and Methods

### 4.1. Chemicals

NAX014 (Figure 1A) was provided by Naxospharma. 13-(4-chlorophenylethyl) berberine iodide (NAX014) was prepared and characterized as previously reported starting from commercial BBR chloride hydrate (ca. 17% H_2_O), which was purchased from Shanghai Trust & We, China [30]. The purity (>97%) of the derivative NAX014 was assessed by HPLC on a Jasco system LC-2000 series (Jasco Europe, Lecco, Italy) with an Agilent Eclipse XDB-C18 (4.6 × 150 mm, 3.5 μm) Rapid Resol column (Agilent Technologies, Santa Clara, CA, USA). The flow rate of the mobile phase (50% water, 50% acetonitrile plus 0.1% trifluoroaceticacid) was maintained at 1 mL/min and absorbance was measured at 235, 265, 340, and 420 nm. The structure was confirmed by HNMR ((200 MHz, DMSO-d6): 10.02 (s, 1H), 9.87 (s, 1H), 9.86 (s, 1H), 8.33 (d, 1H), 8.24 (d, 1H), 7.95 (d, 1H), 7.38 (d, 2H), 7.22 (d, 2H), 7.05 (s, 2H), 6.16 (s, 2H), 4.12 (s, 3H), 4.11 (s, 3H), 4.02 (m, 2H), 3.29 (t, 2H), 2.88 (m, 4H).

### 4.2. Cell lines and Treatments

Human HER-2 overexpressing SK-BR-3 cell line was purchased from IZSLER (Istituto Zooprofilattico Sperimentale della Lombardia e dell’Emilia Romagna “Bruno Ubertini”, Brescia, Italy). Murine TUBO cell line was established in vitro from a carcinoma that spontaneously arose in BALB/c mice carrying the rat HER-2/neu proto-oncogene driven by the mouse mammary tumor virus (MMTV) promoter. SK.BR-3 and TUBO cells were maintained in DMEM medium supplemented with 10% and 20% FBS, respectively, 100 units/mL penicillin and 100 μg/mL streptomycin under a humidified atmosphere of 5% CO_2_ at 37 °C.

### 4.3. Boyden Chamber Cell Chemotaxis Assay

Cell migration assay (Cultrex^®^, Trevigen, Gaithersburg, MD, USA) was used to investigate cell migration following the manufacturer’s instructions. Briefly, cells were grown to 80% confluence and starved in 0.5% FBS medium 24-h before the assay then were added to top chamber (50,000 cells/well), with NAX014 dissolved in DMSO at different concentration (0, 0.5, 2, and 10 μM). The Boyden chamber bottom wells were loaded with 150 μL of medium containing the chemoattractant (20% FBS), and/or vehicle (0.01–0.02% DMSO (*v*/*v*)). The chamber was incubated for 48 h at 37° C. A Cell Dissociation Solution containing Calcein-AM was used for spectrophotometric quantification (485 nm excitation, 520 nm emission) of migrating cells.

### 4.4. Experimental Design

FVB/N HER-2/neu transgenic female mice for the activated rat *neu* oncogene were obtained from Charles River (Hollister, CA, USA) and maintained under specific pathogen-free conditions under a standard 12 h light/12 h dark regimen in our animal facilities. Mice were housed in plastic non-galvanized cages and fed with standard pellet food (Harlan Nossan, Milan, Italy) and tap water ad libitum. The study was conducted in accordance with the ethical standards and according to national and international guidelines and has been approved by the animal research ethics committee of the IRCCS INRCA. Four-month-old mice were randomly divided into two groups which received the following treatments by IG administration for two times a week, at intervals of 3–4 days: (i) 20 mg/kg NAX014 and (ii) 17.7% DMSO (*v*/*v*) in sterile water (control group). Twice a week, all mice were weighed and clinically assessed for signs of toxicity until the end of the study. Mammary tumors growth was monitored by palpation and measured by caliper, then tumor volume was calculated using the formula V = ((length × width^2^)/2). Progressively growing masses of >3 mm in mean diameter were regarded as tumors. Mice were euthanized for ethical reasons when the tumor volume exceeded 500 mm^3^, then tissue collection (blood, tumor masses, and lungs) and ex vivo analysis were performed. The number of lung metastases was evaluated after staining by endotracheal infiltration with 15% china ink solution. Lungs were fixed and bleached in Fekete’s solution (300 mL 70% ethanol, 30 mL 37% formaldehyde, and 5 mL glacial acetic acid). The number of macroscopic pulmonary tumor nodules was counted in each of the five lobes. Diameter of metastatic lesions was measured with calipers and volumes were calculated by 4/3πr^3^, assuming the metastases were spherical. Procedures and facilities followed the requirements of Commission Directive 86/609/EEC concerning the protection of animals used for experimental and other scientific purposes. Italian legislation is defined in D.L. no. 116 of 27 January 1992. The experimental protocols were also approved by the Institutional Animal Care Committee of the Italian Ministry of Health and by the Animal Research Ethics Committee of IRCCS INRCA.

### 4.5. Reverse Transcription, PCR, and Real-Time PCR

Total RNA was isolated from tumor tissues using RNeasy kit (Qiagen, Milan, Italy) according to the manufacturer’s instructions. The first-strand cDNA was synthesized starting from 1 μg of total RNA using RevertAid First Strand cDNA Synthesis Kit (Thermo Fisher Scientific, Waltham, MA, USA). PCR for *perforin*, *granzyme B* and *β-actin* were performed as previously described [31]. Relative quantification of mRNA expression was achieved using fluorescent dye SYBR green (Bio-Rad, Richmond, CA, USA) during PCR amplification (iQ5 Real-time Detection System, Bio-Rad). The primers used were as follows: *β-actin*, 5′-TTCGTTGCCGGTCCACAC-3′, 5′-ACCAGCGCAGCGATATCG-3′; *p16*, 5′-CGTACCCCGATTCAGGTGAT-3′, 5′-TTGAGCAGAAGAGCTGCTACGT-3′; *p53*, 5′-TGGAAGACTCCAGTGGGAAC-3′, 5′-TTTCTTCTTCTGTACGGCGG-3′; *p21*, 5′-GCTGTCTTGCACTCTGGTGT-3′, 5′-TCTGCGCTTGGAGTGATAGA-3′; *p27*, 5′-AACTAACCCGGGACTTGGAG-3′, 5′-CCAGGGGCTTATGATTCTGA-3′; *HER-2*, 5′-ATGTGCTAGTCAAGAGTCCCAAC-3′, 5′-CATCTGCATGGTACTCTGTCTCA-3′. Each set of primers was designed across intron/exon boundaries to detect genomic DNA contamination. Quantitative measurements were determined using the ΔΔCt method and expression of *β-actin* was used as the internal control.

### 4.6. ELISA for Mouse TNF-α and VEGF

Plasma level of Tumor Necrosis Factor-α (TNF-α) and Vascular Endothelial Growth Factor (VEGF) were measured using sandwich ELISA kit according to the manufacturer’s instructions (Boster Biological Technology, Pleasanton, CA, USA).

### 4.7. Flow Cytometric β-Galactosidase Assay

Senescence-associated beta-galactosidase activity (SA-beta-gal) was performed by flow cytometry as previously described [32]. SK-BR-3 cells were grown to 80% confluence in complete medium and then treated with NAX014 using its respective IC85 (inhibitory concentration to kill 85% cells) corresponding to 16.9 μM. DMSO-treated cells have been used as negative control. After 3 days of treatment, the conditioned medium was removed and then replace by prewarmed fresh culture media (washout) for the consecutive four days. Cell viability was determined by trypan blue exclusion assay (Sigma-Aldrich, Merck KGaA, Darmstadt, Germany). The β-Galactosidase assay was performed at each time point following the procedure. Briefly, SK-BR-3 were incubated for one hour in growth medium supplemented with 100 nM Bafilomycin A1 (Sigma-Aldrich, Merck KGaA, Darmstadt, Germany) at 37° C, 5% CO_2_, and two hours in growth medium with 100 nM Bafilomycin A1 and 33 μM C_12_FDG (Invitrogen, Thermo Fisher Scientific, Waltham, MA, USA) before analysis. After incubation, cells were trypsinized, washed with PBS, resuspended in 200 μL ice-cold PBS, and analyzed using a Coulter Epics XL flow cytometer. Data were analyzed with the instrument software, and cell debris was excluded on basis of light scatter parameters. C_12_FDG was measured on the FL1 median fluorescence intensity (MFI) of the SK-BR-3 population.

### 4.8. Statistical Analysis

All statistical analyses were conducted using SPSS 20 (IBM, Armonk, NY, USA). Kaplan-Meier analysis was performed to generate the tumor-free curves. Statistical inference was done with the log-rank test to compare survival curves across groups. The Pearson χ2 test was used for the number of metastatic sites comparison. In order to evaluate the magnitude of the fold change data (between groups) referred to the mRNA expression, the effect size (Cohen’s d) was determined.

## Data Availability

The data presented in this study are available on request from the corresponding author.

## Supplementary Materials

The following are available online at https://www.mdpi.com/1422-0067/22/5/2653/s1, Figure S1: Effect of NAX014 administration on tumor number and tumor volume in FVB/N HER-2/neu transgenic mice, Figure S2: Effect of NAX014 administration on *granzyme B* and *perforin* mRNA expression in tumor masses.

## References

[1] Rimawi M.F., Schiff R., Osborne C.K. (2015). Targeting HER2 for the treatment of breast cancer. Annu. Rev. Med..

[2] Rexer B.N., Arteaga C.L. (2012). Intrinsic and acquired resistance to HER2-targeted therapies in HER2 gene-amplified breast cancer: Mechanisms and clinical implications. Crit. Rev. Oncog..

[3] Seidman A., Hudis C., Pierri M.K., Shak S., Paton V., Ashby M., Murphy M., Stewart S.J., Keefe D. (2002). Cardiac Dysfunction in the Trastuzumab Clinical Trials Experience. J. Clin. Oncol..

[4] Ortega M.A., Fraile-Martínez O., Guijarro L.G., Casanova C., Coca S., Álvarez-Mon M., Buján J., García-Honduvilla N., Asúnsolo Á. (2020). The Regulatory Role of Mitochondrial MicroRNAs (MitomiRs) in Breast Cancer: Translational Implications Present and Future. Cancers.

[5] Pallerla S., Abdul A.U.R.M., Comeau J., Jois S. (2021). Cancer vaccines, treatment of the future: With emphasis on her2-positive breast cancer. Int. J. Mol. Sci..

[6] Gupta B.M. (1930). Das The Treatment of Oriental Sore with Berberine Acid Sulphate. Ind. Med. Gaz..

[7] Tillhon M., Guamán Ortiz L.M., Lombardi P., Scovassi A.I. (2012). Berberine: New perspectives for old remedies. Biochem. Pharmacol..

[8] Pierpaoli E., Arcamone A.G., Buzzetti F., Lombardi P., Salvatore C., Provinciali M. (2013). Antitumor effect of novel berberine derivatives in breast cancer cells. BioFactors.

[9] Wang Y.X., Liu L., Zeng Q.X., Fan T.Y., Jiang J.D., Deng H.B., Song D.Q. (2017). Synthesis and identification of novel berberine derivatives as potent inhibitors against TNF-α-induced nf-κb activation. Molecules.

[10] Guamán Ortiz L.M., Tillhon M., Parks M., Dutto I., Prosperi E., Savio M., Arcamone A.G., Buzzetti F., Lombardi P., Scovassi A.I. (2014). Multiple effects of berberine derivatives on colon cancer cells. Biomed. Res. Int..

[11] Pierpaoli E., Damiani E., Orlando F., Lucarini G., Bartozzi B., Lombardi P., Salvatore C., Geroni C., Donati A., Provinciali M. (2015). Antiangiogenic and antitumor activities of berberine derivative NAX014 compound in a transgenic murine model of HER2/neu-positive mammary carcinoma. Carcinogenesis.

[12] Abrams S.L., Follo M.Y., Steelman L.S., Lertpiriyapong K., Cocco L., Ratti S., Martelli A.M., Candido S., Libra M., Murata R.M. (2019). Abilities of berberine and chemically modified berberines to inhibit proliferation of pancreatic cancer cells. Adv. Biol. Regul..

[13] Kheir M.M., Wang Y., Hua L., Hu J., Li L., Lei F., Du L. (2010). Acute toxicity of berberine and its correlation with the blood concentration in mice. Food Chem. Toxicol..

[14] Butler M.S., Robertson A.A.B., Cooper M.A. (2014). Natural product and natural product derived drugs in clinical trials. Nat. Prod. Rep..

[15] Bhowmik D., Buzzetti F., Fiorillo G., Orzi F., Syeda T.M., Lombardi P., Suresh Kumar G. (2014). Synthesis of new 13-diphenylalkyl analogues of berberine and elucidation of their base pair specificity and energetics of DNA binding. Medchemcomm.

[16] Pierpaoli E., Fiorillo G., Lombardi P., Salvatore C., Geroni C., Piacenza F., Provinciali M. (2018). Antitumor activity of NAX060: A novel semisynthetic berberine derivative in breast cancer cells. BioFactors.

[17] Milata V., Svedova A., Barbierikova Z., Holubkova E., Cipakova I., Cholujova D., Jakubikova J., Panik M., Jantova S., Brezova V. (2019). Synthesis and anticancer activity of novel 9-O-substituted berberine derivatives. Int. J. Mol. Sci..

[18] Eek D., Krohe M., Mazar I., Horsfield A., Pompilus F., Friebe R., Shields A.L. (2016). Patient-reported preferences for oral versus intravenous administration for the treatment of cancer: A review of the literature. Patient Prefer. Adherence.

[19] Kennecke H., Yerushalmi R., Woods R., Cheang M., Voduc D., Speers C., Nielsen T., Gelmon K. (2010). Metastatic behavior of breast cancer subtypes. J. Clin. Oncol..

[20] Molnár I.A., Molnár B.Á., Vízkeleti L., Fekete K., Tamás J., Deák P., Szundi C., Székely B., Moldvay J., Vári-Kakas S. (2017). Breast carcinoma subtypes show different patterns of metastatic behavior. Virchows Arch..

[21] Xiao W., Zheng S., Liu P., Zou Y., Xie X., Yu P., Tang H., Xie X. (2018). Risk factors and survival outcomes in patients with breast cancer and lung metastasis: A population-based study. Cancer Med..

[22] Weis S.M., Cheresh D.A. (2005). Pathophysiological consequences of VEGF-induced vascular permeability. Nature.

[23] Leibovich S.J., Polverini P.J., Shepard H.M., Wiseman D.M., Shively V., Nuseir N. (1987). Macrophage-induced angiogenesis is mediated by tumour necrosis factor-α. Nature.

[24] Bando H., Weich H.A., Brokelmann M., Horiguchi S., Funata N., Ogawa T., Toi M. (2005). Association between intratumoral free and total VEGF, soluble VEGFR-1, VEGFR-2 and prognosis in breast cancer. Br. J. Cancer.

[25] Sheen-Chen S.M., Chen W.J., Eng H.L., Chou F.F. (1997). Serum concentration of tumor necrosis factor in patients with breast cancer. Breast Cancer Res. Treat..

[26] Spatuzza C., Postiglione L., Covelli B., Ricciardone M., Benvenuti C., Mondola P., Belfiore A. (2014). Effects of berberine and red yeast on proinflammatory cytokines IL-6 and TNF-α in peripheral blood mononuclear cells (PBMCs) of human subjects. Front. Pharmacol..

[27] Pierpaoli E., Cirioni O., Simonetti O., Orlando F., Giacometti A., Lombardi P., Provinciali M. (2020). Potential application of berberine in the treatment of Escherichia coli sepsis. Nat. Prod. Res..

[28] Lee S., Lee J. (2019). Cellular senescence: A promising strategy for cancer therapy. BMB Rep..

[29] Short S., Fielder E., Miwa S., von Zglinicki T. (2019). Senolytics and senostatics as adjuvant tumour therapy. EBioMedicine.

[30] Lombardi P., Buzzetti F., Arcamone A. (2012). Benzoquinolizinium Salt Derivatives as Anticancer Agents. U.S. Patent.

[31] Anisimov V.N., Berstein L.M., Egormin P.A., Piskunova T.S., Popovich I.G., Zabezhinski M.A., Kovalenko I.G., Poroshina T.E., Semenchenko A.V., Provinciali M. (2005). Effect of metformin on life span and on the development of spontaneous mammary tumors in HER-2/neu transgenic mice. Exp. Gerontol..

[32] Noppe G., Dekker P., De Koning-Treurniet C., Blom J., Van Heemst D., Dirks R.W., Tanke H.J., Westendorp R.G.J., Maier A.B. (2009). Rapid flow cytometric method for measuring senescence associated β-galactosidase activity in human fibroblasts. Cytom. Part A.

