# Supplementary material for: Antimetastatic and Antitumor Activities of Orally Administered NAX014 Compound in a Murine Model of HER2-Positive Breast Cancer
Source: Int J Mol Sci. 2021 Mar 6;22(5):2653. doi: 10.3390/ijms22052653 (PMC7961369; doi:10.3390/ijms22052653)
Supplement: Supplementary file 1 [file ijms-22-02653-s001.pdf]

Supplementary Figures

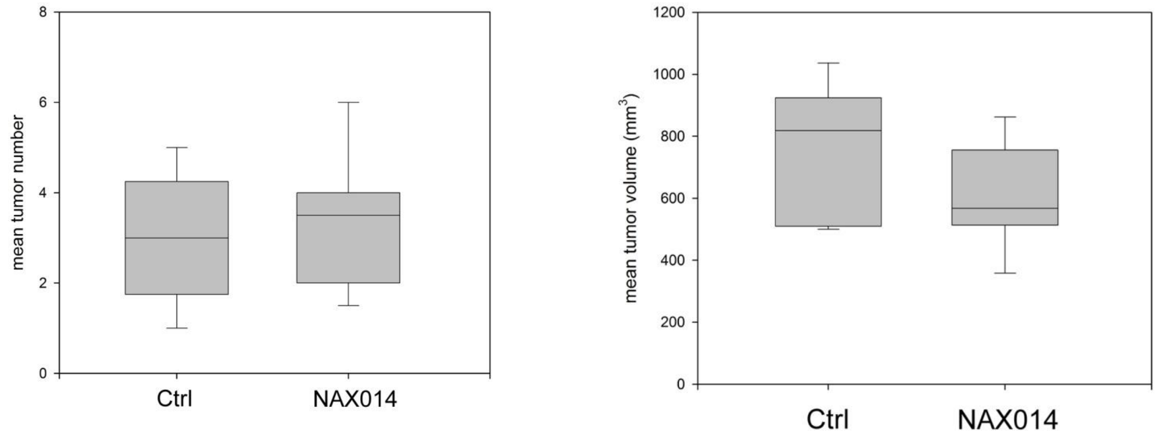

Figure S1. Effect of NAX014 administration on tumor number and tumor volume in FVB/N HER-2/neu transgenic mice. The mean tumors number *per* mouse (left panel) was calculated as the cumulative number of incident tumors at the time of suppression (500 mm^3). Graph in the right panel represents the overall tumors volume/mouse at the time of suppression (500 mm^3). Statistical analysis was assessed using a univariate analysis of variance (ANOVA) with a Student–Newman–Keuls post hoc test. $p > 0.05$ compared with control group.

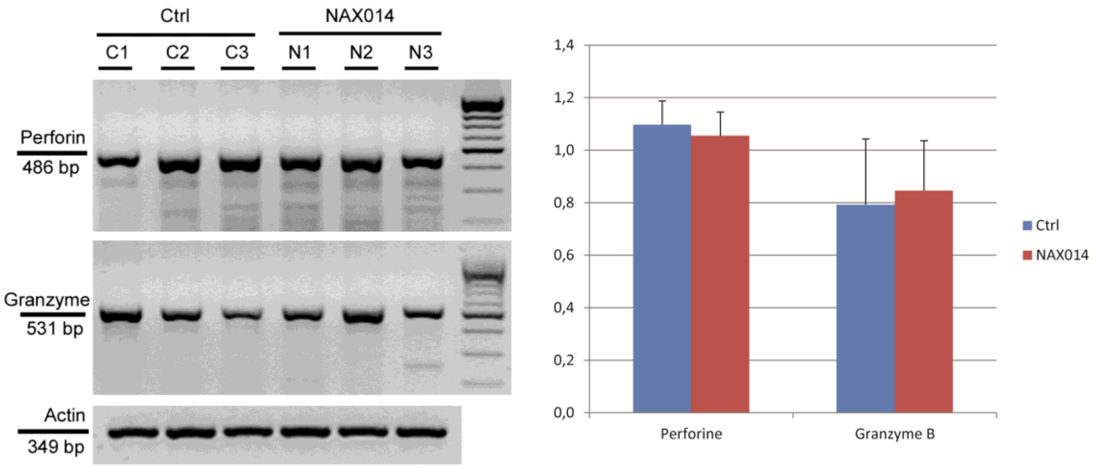

Figure S2. Effect of NAX014 administration on *granzyme B* and *perforin* mRNA expression in tumor masses. Expression analysis was performed through conventional PCR and following by densitometric quantification. Graphs represent the mean±SD of *β-actin*-normalized values. $p > 0.05$ compared with control values.
